# Transurethral prostate thermotherapy: filtering the bowel motion during MR-thermometry processing

**DOI:** 10.1186/2050-5736-3-S1-O61

**Published:** 2015-06-30

**Authors:** Alain Schmitt, Charles Mougenot, Rajiv Chopra

**Affiliations:** 1 Sunnybrook Research Institute, Toronto, Canada; 2 Philips Healthcare Canada, Toronto, Canada; 3 University of Texas Southwestern Medical Center, Dallas, Texas, United States

## Background/introduction

Magnetic resonance imaging (MRI)-guided transurethral ultrasound therapy is a minimally-invasive image-guided treatment for localized prostate cancer offering precise targeting of tissue within the gland. The accuracy of MR thermometry is critical for precise on-line feedback control as well as for monitoring potential thermal injury of surrounding tissues, especially the rectum wall. The PRFS thermometry method used is sensitive to tissue motion and change in the local magnetic susceptibility which might be related to the motion of air bubbles in the rectum in the particular case of MR controlled prostate cancer therapy.

## Methods

This method aims to filter sudden temperature artifacts. The filter is based on the analysis of the temporal standard deviation of the temperature relatively to the measurement noise and ultrasonic heating distribution. Inconsistent temperature variations are detected and cancelled in a first step. As a second step, a spatial averaging using reliable voxels is applied in this artifacted region to keep consistent heating distribution and maintain a low noise level.

## Results and conclusions

This filter has been evaluated by post-processing data from five human transurethral ultrasound treatments. The two-steps correction of the artifact detected areas reduces the final standard deviation to levels similar to the originally non-artifacted prostate and rectum areas. Evaluation of the filter on the patient data showed that most artifacts due to the presence of moving air bubbles in the rectum have been detected and removed. A quantitative estimation of the filter capabilities shows a systematic improvement in the standard deviation of the corrected temperature maps, up to 2.2°C improvement on the artifacted rectum zone.

**Figure 1:**
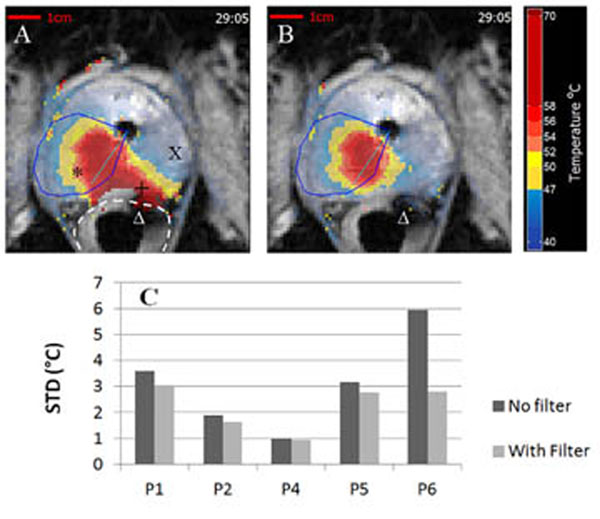
MR thermal maps without (A) and with filter (B), during transurethral ultrasound therapy with moving air bubble in the rectum (Δ) inducing a temperature artifact (+) that interfered with the real heating (*). Average STD over the Rectum zone by patient.

